# Bridging the Molecular-Cellular Gap in Understanding Ion Channel Clustering

**DOI:** 10.3389/fphar.2019.01644

**Published:** 2020-01-29

**Authors:** Valerie Abigail Nirenberg, Ofer Yifrach

**Affiliations:** Department of Life Sciences and the Zlotowski Center for Neurosciences, Ben-Gurion University of the Negev, Be’er Sheva, Israel

**Keywords:** action potential, clustering, coupling, ion channel density, potassium channels, sodium channels, post-synaptic density–95, scaffold proteins

## Abstract

The clustering of many voltage-dependent ion channel molecules at unique neuronal membrane sites such as axon initial segments, nodes of Ranvier, or the post-synaptic density, is an active process mediated by the interaction of ion channels with scaffold proteins and is of immense importance for electrical signaling. Growing evidence indicates that the density of ion channels at such membrane sites may affect action potential conduction properties and synaptic transmission. However, despite the emerging importance of ion channel density for electrical signaling, how ion channel-scaffold protein molecular interactions lead to cellular ion channel clustering, and how this process is regulated are largely unknown. In this review, we emphasize that voltage-dependent ion channel density at native clustering sites not only affects the density of ionic current fluxes but may also affect the conduction properties of the channel and/or the physical properties of the membrane at such locations, all changes that are expected to affect action potential conduction properties. Using the concrete example of the prototypical *Shaker* voltage-activated potassium channel (Kv) protein, we demonstrate how insight into the regulation of cellular ion channel clustering can be obtained when the molecular mechanism of ion channel-scaffold protein interaction is known. Our review emphasizes that such mechanistic knowledge is essential, and when combined with super-resolution imaging microscopy, can serve to bridge the molecular-cellular gap in understanding the regulation of ion channel clustering. Pressing questions, challenges and future directions in addressing ion channel clustering and its regulation are discussed.

## Introduction

The precise localization, distribution, and density of voltage-activated ion channels at specific neuronal membrane sites are essential for action potential (AP) generation and propagation and for synaptic transmission (Hille, 1992). Ion channels are not randomly distributed in the membrane, as would be assumed by the Singer-Nicolson fluid mosaic membrane model (Singer and Nicolson, 1972), but instead are targeted to specific sites, such as axon initial segments (AIS), nodes of Ranvier (NR) or the post-synaptic density (PSD), where they are usually part of highly regulated multi-protein macromolecular complexes (Lai and Jan, 2006). Transcriptional activators and suppressors, cytoskeletal proteins, cell adhesion molecules, post-translational signaling molecules, ion channel auxiliary subunits, and scaffold proteins are co-targeted with ion channels to such sites. The final endpoint in assembling such complexes is not only the proximity of the ion channel and its auxiliary subunits and/or modulatory proteins but also the clustering of many ion channel molecules together, potentially, within interaction distances (Engelman, 2005; Han and Kim, 2008; Freeman et al., 2016; Capera et al., 2019). The density of ion channel molecules at these sites, i.e., the number of molecules per membrane area, is thus an important parameter, and the question then arises what are the potential functional consequences of having many neighboring ion channel molecules aggregated together in one site with respect to electrical signaling? Furthermore, how the density of ion channel proteins at their specific membrane targeting sites are spatially and temporally regulated, to affect AP conduction properties, is largely unknown.

In the current review, we highlight the importance of ion channel clustering and its regulation for electrical signaling. We begin by concisely surveying where and how different voltage-activated ion channels are targeted to primary sites along the polarized neuron in the direction of AP propagation. We then present cases where ion channel density was shown to affect not only the density of ionic current fluxes at their site of expression but also the gating properties of ion channels, relative to their function in an isolated context, and the physical properties of the membrane. We discuss the functional consequences of such changes on AP conduction properties and further emphasize the important role played by scaffold proteins in mediating voltage-dependent ion channel clustering. Finally, by using the concrete example of the voltage-activated potassium channel protein, we highlight that when the molecular mechanism underlying the ion channel-scaffold protein interaction is known, cellular understanding regarding the regulation of ion channel density can be obtained. This example demonstrates the strength of adopting a mechanistic view in bridging the molecular-cellular gap in the understanding of ion channel clustering. We end with a summary of current challenges and consider future directions that can be taken for better understanding ion channel clustering.

## Site-Specific Clustering of Ion Channels Affects Signal Generation, Propagation, and Transmission

**Figure 1:**
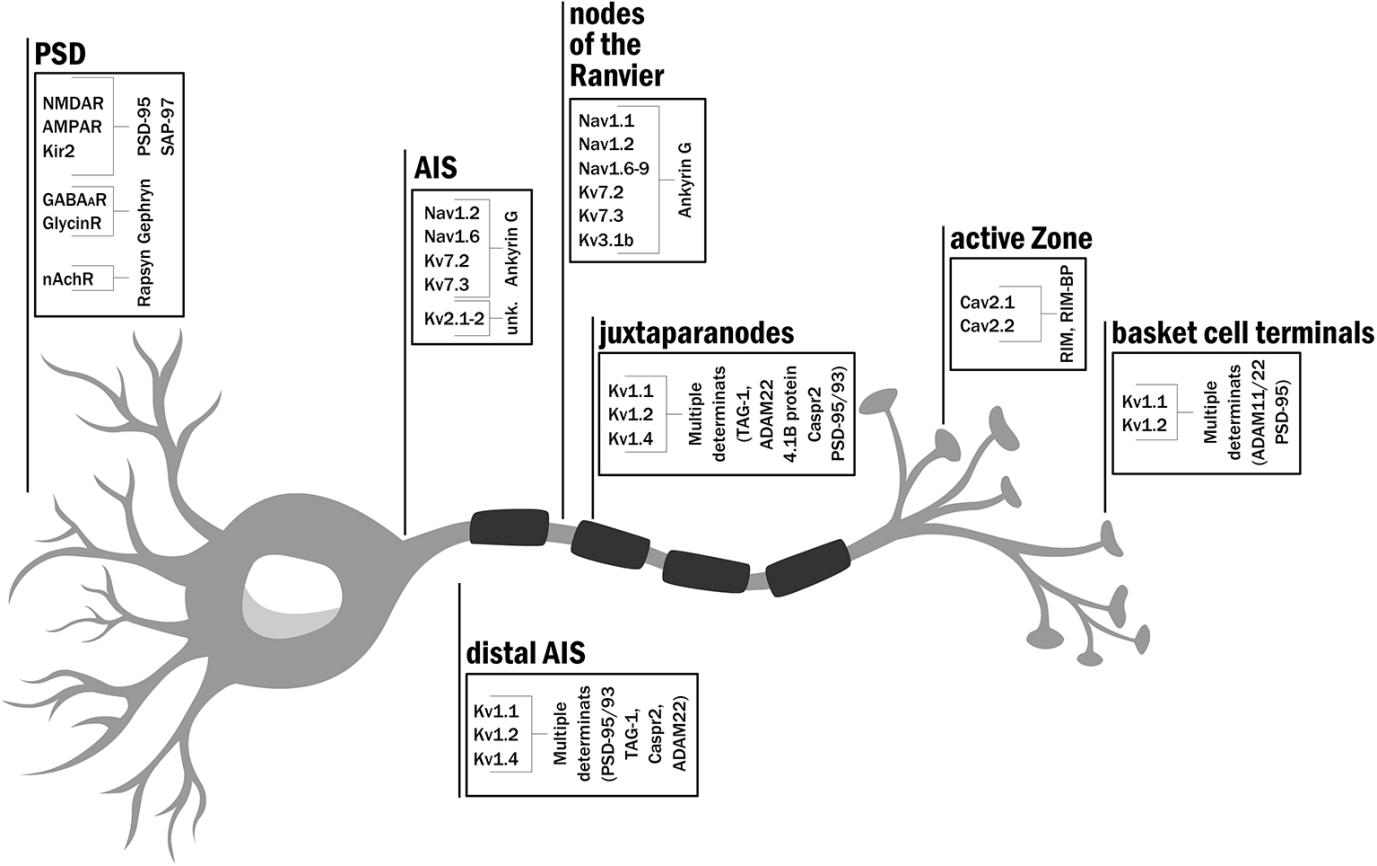
Clustering geography: the distribution of ion channels and scaffold proteins along the neuron. Schematic representation of a typical neuron specifying the type of ion channel and scaffold proteins targeted and clustered to the axon initial segment, node of Ranvier, active zone and at the postsynaptic densities (in dendrites). A full reference list supporting the indicated location for each channel subtype is given in **SI Text 1**.

Action potentials are transient changes in membrane polarization that spread in space and time along neurons or muscle cells, for example. Such perturbations result from choreographed changes in membrane conductance, primarily by Na^+^ and K^+^ ions, brought about by opening, closing, and inactivation gating transitions of voltage-activated sodium (NaV) and potassium (Kv) ion channels, respectively. One can encounter clusters of the various voltage-dependent ion channels starting at the neuronal soma, continuing along the axon and across the synapse (**Figure 1**). AP generation relies on proper enrichment of Na^+^ and K^+^ voltage-gated ion channels to the AIS, where the axon begins to extend from the soma (Hille, 1992; Kole et al., 2007; Kole et al., 2008; Jensen et al., 2017). Membrane depolarization occurs with high efficiency at the AIS, with voltage-activated sodium channel subtypes 1.2 and 1.6 (NaV1.2 and NaV1.6) corresponding to the dominant ion channel types clustered at this region (Tian et al., 2014) (**Figure 1**). Disruption of NaV channel cluster formation at the AIS in mouse cerebellum Purkinje cells led to delayed AP initiation and a reduced maximal firing rate (Zhou et al., 1998). These effects emphasize the importance of NaV channel clusters at the AIS for normal electrical signaling. Kv channels were also shown to be part of the high-density channel repertoire at the AIS (**Figure 1**). While Kv2.1 channel clusters are seen at specific subdomain patches of the AIS (Trimmer, 1991; Scannevin et al., 1996; Sarmiere et al., 2008), Kv7, and Kv1 channel subtypes (KCNQ and *Shaker*-type channels), respectively, reside at the proximal (Klinger et al., 2011) and distal regions of the AIS (Kole et al., 2007), adjusting the threshold, inter-spike interval, and firing frequency characteristics of the action potential.

As the AP propagates down the myelinated axon, high densities of NaV and Kv channels are encountered at nodes of the Ranvier (**Figure 1**). Here, targeting and clustering of these channels are essential for AP saltatory conduction along the axon (Rasband and Trimmer, 2001). The nodal membrane was reported to express clusters of multiple NaV channel subtypes, including NaV1.1, NaV1.2, NaV1.6, NaV1.7, Nav1.8, and NaV1.9 channels (Chang and Rasband, 2013). Kv channel subtypes 1, 3, and 7 were also reported to reside and cluster at nodal sites. Whereas Kv7.2, Kv7.3, and Kv3.1 channels were shown to be responsible for the repolarization of the axonodal membrane (Devaux et al., 2003; Devaux et al., 2004; Klinger et al., 2011; Trimmer, 2015), the Kv1 channel subfamily (Kv1.1, Kv1.2, and Kv1.4) is clustered at juxtaparanodal regions (Poliak et al., 2003; Rasband, 2004) and serves to adjust AP propagation along the myelinated axon. Pharmacologically blocking these channels resulted in prolonged action potentials (Devaux et al., 2002).

Progressing along the axon towards its terminal, one reaches the active zone (AZ), the next site where ion channels are clustered (**Figure 1**). In this region, proteins of the vesicle release machinery involved in synaptic transmission are tightly docked (Südhof, 2012). In particular, voltage-activated Ca^2+^ channels (N and P/Q types, specifically CaV2.1 and 2.2, respectively) are clustered at this site to ensure the economic and directed release of neurotransmitters into the synaptic cleft (Kaeser et al., 2011; Gundelfinger and Fejtova, 2012). Following the release, neurotransmitters diffuse across the synaptic cleft and bind to excitatory or inhibitory ligand-gated ionotropic receptors that are aggregated at the post-synaptic density membrane (PSD). The PSD has mostly been studied in glutamatergic neurons, where AMPA and NMDA ionotropic receptors are clustered, proximal to the pre-synaptic AZ sites (Boeckers, 2006; Chen et al., 2015; Tang et al., 2016; Scheefhals and MacGillavry, 2018). Ionotropic glycine receptor clustering was also reported in the membrane of the postsynaptic cell (Patrizio et al., 2017; Schaefer et al., 2018). Such clusters are found in motor neuronal membranes of the adult spinal cord, brain stem, and retina, and were shown to be essential for proper neuron hyperpolarization. Impaired clustering of glycine receptors is associated with startle disease in mice (Patrizio et al., 2017; Schaefer et al., 2018). The clustering of different ion channels and receptors in proximity to other intracellular signaling and cytoskeletal structural proteins at the PSD is also essential for ensuring a robust response to neurotransmitter binding and for modulating the evoked synaptic potential, whether inhibitory or excitatory, of the post-synaptic cell membrane (Boeckers, 2006). A robust response to neurotransmitter release also occurs at the neuromuscular junction (NMJ) where it is essential for muscle contraction. Thousands of copies of nicotinic acetylcholine receptors (nAchRs) are clustered at the post-synaptic muscle cell (Peng and Poo, 1986; Bruneau et al., 2008). The opening of these non-selective ligand-gated cation channels in response to acetylcholine release allows the diffusion of primarily Na^+^ ions across the muscle cell membrane to achieve efficient muscle cell membrane depolarization and subsequently contraction (Bruneau et al., 2008; Slater, 2017; Burden et al., 2018). A detailed reference list summarizing available information attesting to the targeting of a specific ion channel subtype to the indicated neuronal membrane sites is given in **SI Text 1**.

## The Hidden Dimension of Ion Channel Clustering: the Different Modes by Which Ion Channel Density May Affect AP Conduction Properties

The density of aggregated voltage-dependent ion channel molecules at their unique sites of targeting may affect AP conduction properties *via* three distinct, non-mutually-exclusive modes. First and straightforward, voltage-dependent ion channel density at the sites of channel expression directly dictates the density of inward or outward Na^+^ or K^+^ ionic currents, upon changes in membrane polarization. Second, ion channel density by itself may affect activation and/or inactivation gating transitions of the channel, as compared to the isolated channel scenario. Last, the accumulation of densely-packed voltage-activated ion channel molecules, with their multiple positively charged voltage-sensing domains, might affect the capacitance property of the membrane at such sites, potentially affecting the cable properties of the axon. As we concisely summarize below, all three functional consequences of changes in ion channel density are expected to affect AP conduction properties.

Several years before the seminal papers of Hodgkin and Huxley, describing the theory behind action potential generation (Hodgkin and Huxley, 1952; Huxley, 1964), Hodgkin described a set of experiments on *Carcinus maenas* crab axons demonstrating two, low- and high-frequency, repetitive firing modes (Hodgkin, 1948). These experiments provided a solid base for the current understanding of AP firing modes exhibited by pyramidal neurons (Tateno and Robinson, 2006) and interneurons (Tateno and Robinson, 2007). The two neuronal excitability modes were used to rationalize coding properties of neurons (Rinzel and Ermentrout, 1998; St-Hilaire and Longtin, 2004; Tateno et al., 2004; Tateno and Robinson, 2006; Tateno and Robinson, 2007). It has long been thought that different combinations of ion channel types may explain the observed differences in firing patterns (Prescott et al., 2008). In recent years, however, theoretical and experimental evidence has been accumulated to show that changes in the densities of voltage-gated Na^+^ and K^+^ channels alone can explain the dynamic switch of a neuron between the low and high-frequency firing modes (Århem et al., 2006; Århem and Blomberg, 2007; Zeberg et al., 2010; Zeberg et al., 2015). Such changes in ionic current densities are reflected by changes in the membrane permeability to Na^+^ and K^+^ ions when all channels are open. In the native channel context, such modulation of ion channel densities may be brought about by specific channel blockers that serve to reduce the nominal number of active channels per site (Zeberg et al., 2010). These later studies directly demonstrate how changes in ionic current densities may affect AP conduction properties and information coding.

Changes in ion channel densities at their native clustering sites may further affect the gating properties of the ion channel. Ion channel gating is often studied in an isolated channel context, with the ion channel being expressed in the membrane of a heterologous expression system (Furutani and Kurachi, 2012). Activation and slow or fast channel inactivation of voltage-dependent channels are then studied using electrophysiological recording techniques, with the single-channel recording being one of the most direct and thorough methods for studying ion channel gating and regulation (Sakmann and Neher, 1984). Clearly, the targeting of ion channels to native sites along with their adjacent modulatory subunits and regulatory proteins affects the gating properties of the channels (O’Connell et al., 2010; Zhang et al., 2016; Vivas et al., 2017). What we wish to emphasize here is that the density of ion channels at native clustering sites by itself may affect the channel activation and inactivation gating properties, thus affecting AP conduction properties. This dimension is frequently overlooked when addressing channel clustering. Several examples of such density-mediated modulation of channel gating have been reported. First, the density of the prototypical bacterial KcsA K^+^ channel expressed in liposomes was shown to affect the open probability of the channel (Molina et al., 2006; Sumino et al., 2014). The mechanism underlying such effects on channel activation gating is, however, not yet clear. Another example is the L-type CaV1.2 channel expressed in native cardio-myocyte cells. CaV1.2 channels were shown reside within small clusters (5–10 molecules) and appear to open synchronously in response to membrane depolarization (Dixon et al., 2015). Furthermore, prolonged open channel duration was observed for the clustered channels, as compared to the isolated channel case, suggesting that activation gating is affected (Dixon et al., 2015; Folci et al., 2018). The functional coupling between multiple ion channels can also be inhibitory. For instance, Kv2.1 channels were reported to exhibit a non-conductive phenotype when clustered at high densities (Scannevin et al., 1996; O’Connell et al., 2010; Fox et al., 2013; Liu et al., 2016). Clearly, in all these examples, although no mechanisms were suggested, it is reasonable to assume that short-range inter-molecular allosteric interactions are responsible for the observed coupling effects. Such changes in ion channel gating properties due to changes in channel density would affect the kinetics of ionic current development with time (i.e. the rate constants for activation and inactivation channel processes) and are expected to affect AP shape and conduction properties (Debanne et al., 1997; Giese et al., 1998; Johnston et al., 1999).

Another dimension of voltage-dependent ion channel density concerns their potential effect(s) on the physical properties of the membrane at the clustering site. Such effects may be brought about by the proximity of many charged voltage sensor domains resulting in changes in membrane thickness and/or its effective dielectric constant, both properties that affect membrane capacitance. The first report concerning potential effects of channel overexpression on membrane properties concerned the channelrhodopsin-2 light-gated ion channel (Zimmermann et al., 2008). Overexpression of the protein in HEK293 cells resulted in changes in the morphology of the cell mabrane reflected in an increase in membrane capacitance. This effect was not observed upon physiological expression levels of the channel (Zimmermann et al., 2008). Furthermore, recordings from tsA201 and PC12 cells overexpressing *the Ciona intestinalis* voltage-sensing phosphatase protein (Ci-VSP) revealed an increase of the local membrane capacitance upon voltage activation of the protein (Hossain et al., 2008; Lundby et al., 2008). The additional capacitance component termed “sensing capacitance” was further investigated by computational simulations of neuronal model cells expressing the voltage-sensing fluorescent proteins VSFP2.3 and VSFP3.1 (Akemann et al., 2009). The results revealed that changes in expression levels of these proteins resulted in changes in membrane capacitance due to changes membrane topology and/or mobility of channel gating, leading to changes in the effective diaelectric constant of the membrane. The simulations revealed that such changes lead to an increase of the threshold for AP spike initiation (Akemann et al., 2009). Although these studies relate to changes in the expression levels of these voltage-sensing domain-containing proteins as causing changes in membrane capacitance, such changes, are of course, related to changes in the density of these proteins per membrane area. The end result is that for voltage-sensor containing proteins, regulation of protein clustering affects membrane capacitance properties and hence, cable properties of the axon. It remains to be shown whether such “sensing capacitance” effects are also observed for native voltage-dependent sodium and potassium ion channel proteins.

Taken together, the results summarized here emphasize that understanding of how ion channel density within clustering sites is regulated is of paramount importance in considering electrical signaling, with changes in ion channel density exerted through changes in membrane ionic current densities, in channel gating properties and/or membrane capacitance, dramatically affecting AP shape, frequency and information coding in neurons.

## Molecular and Cellular Gaps in Understanding Ion Channel Clustering

The family of scaffold proteins is central for targeting and clustering of ion channels at native membrane sites (Good et al., 2011). Such clustering can be revealed by confocal light microscopy imaging of heterologous cells transfected to express both the ion channel and scaffold proteins and is manifested as a speckled co-localization pattern of fluorescently-tagged versions of both proteins in the cell membrane (Tejedor et al., 1997). Scaffold proteins correspond to many different and structurally unrelated protein subfamilies that seem to share similar functional roles. They are multi-domain proteins that interact with ion channels and membrane receptors (as well as with other membrane-embedded proteins) on the one hand, and with other intracellular signaling and skeletal proteins on the other. Scaffold proteins thus link membrane-associated events with downstream signaling pathways (Good et al., 2011; Zheng et al., 2011; Lee et al., 2014).

Here, we consider several examples of scaffold proteins encountered along the length of the neuron (**Figure 1**). Ankyrin G scaffold proteins interact with NaV channel isoforms at nodal and AIS sites and further bind to the intra-cellular βIV-spectrin protein (Zhou et al., 1998; Komada and Soriano, 2002). This latter protein further interacts with actin, a major component of the cytoskeletal network. The interaction between ankyrin G and NaV channels in the rat is mediated by a nine amino acid motif within the second intracellular loop of the NaV1.2 channel and leads to NaV channel clustering (Lemaillet et al., 2003). How ankyrin G serves to dock and cluster NaV channel molecules is not yet known. The clustering of calcium channels at the pre-synaptic AZ offers another example. Here, the scaffold protein of focus is the RIM (Rab3-interacting molecule) protein (Südhof, 2012). RIM proteins were proposed to interact *via* their PDZ protein-protein interaction modules with a cytoplasmic C-terminal PDZ-binding motif presented by N and P/Q type voltage-activated Ca^2+^ channels (Kaeser et al., 2011). Such interaction allows for tethering these channels at the AZ in high copy numbers. For example, around 100 CaV1.3 and ~10 CaV2.1 channel molecules reside in AZ clusters of hippocampal Purkinje neurons, with several such clusters being noted per AZ (Miki et al., 2017). RIM proteins also interact with several other vesicle-release proteins in the AZ complex (Krinner et al., 2017). The RIM scaffold protein thus mediates coupling between the CaV channel and vesicle docking machinery proteins and is essential for synchronous neurotransmitter release at the AZ. Still, it remains to be determined whether or not CaV channel-RIM protein-protein interactions affect Cav gating. The last example concerns the scaffold protein-mediated clustering of different ion channels and receptors at the PSD (Gomperts, 1996). Here, the dominating scaffold protein family is the membrane-associated guanylate kinase (MAGUK) family, the most intensively studied of all scaffold proteins. Relying on their multiple domains, MAGUKs serve as linker proteins that couple membrane events with downstream intracellular signaling (Zheng et al., 2011; Chen et al., 2015). Similar to RIMs, MAGUK proteins also carry PDZ domains capable of binding the C-terminal motifs of several membrane-embedded ion channels, receptors, and cell adhesion proteins (Li et al., 2000; Lee and Zheng, 2010). Other modular domains of MAGUKs, such as its SH3 and guanylate kinase-like domains, serve as hubs for signaling control and interact with other PSD components, such as the adhesion protein ADAM22, A-kinase anchor proteins (AKAPs), actin dynamic modulators (such as SPIN90/WISH) (Lee et al., 2006; Han and Kim, 2008; Verpelli et al., 2012; Frank and Grant, 2017) and other scaffolding proteins [such as *guanylate kinase-associated protein (*GKAP) family members]. PSD-95 and SAP-97, two members of the MAGUK family of scaffold proteins, were shown to be responsible for AMPAR, NMDAR and Kir2 channel clustering at the PSD site of glutamatergic synapses (Cohen et al., 1996; Gomperts, 1996; Chen et al., 2015; Hoffmann et al., 2015). PSD-95 and SAP-97 were also shown to interact with members of the Kv1 (*Shaker*-type) subfamily (Gomperts, 1996; Tiffany et al., 2000). PSD-95 and PSD-93 were also reported to be essential for the proper clustering of *Shaker* channels at the AIS (Ogawa et al., 2008), at membranes of basket cell terminals (Rasband, 2010) and in smooth muscle cells (Joseph et al., 2011) but not in the juxtaparanodes (Rasband et al., 2002). Although Kv1 channels and PSD-95 co-localize at the juxtaparanode (Rasband, 2004; Arancibia-Carcamo and Attwell, 2014), clustering does not seem to depend on PSD-95 and as such, its role in the juxtaparanodal scaffold is still questioned. A detailed reference list supporting the findings indicated above is provided in **SI Text 1**.

The examples above, and others not addressed here demonstrate that ion channel clustering at distinct neuronal membrane sites primarily involves the direct binding of the channel to its cognate scaffold protein partner. In some cases, the ion channel and the scaffold protein binding partners are known. However, how this molecular binding event leads to cellular ion channel clustering involving many molecules is utterly unknown for almost all ion channel proteins. Furthermore, we generally do not know if and how ion channel membrane density is regulated in the spatial and/or temporal dimensions. These questions reflect what we refer to as a molecular-cellular gap that hampers our understanding of ion channel clustering. Indeed, many questions concerning the scaffold protein–ion channel interacting pair have yet to be answered. What is the stoichiometry of the interaction? How is ion channel density regulated in both the spatial and temporal dimensions? How is cluster area size determined? How do other proteins serve to regulate the clustering process? How many ion channel molecules usually reside in such clusters? Are there direct inter-molecular channel–channel interactions within clusters? To begin answering these questions, insight into the molecular mechanism underlying the binding of a channel to its cognate scaffold protein must be obtained. As will be demonstrated in the following section using the concrete example of the *Shaker* Kv channel-PSD-95 interaction, where such mechanistic knowledge is available, valuable insight into the mechanism of ion channel clustering is at hand.

## Bridging the Molecular-Cellular Gap in Understanding Kv Channel Clustering

**Figure 2:**
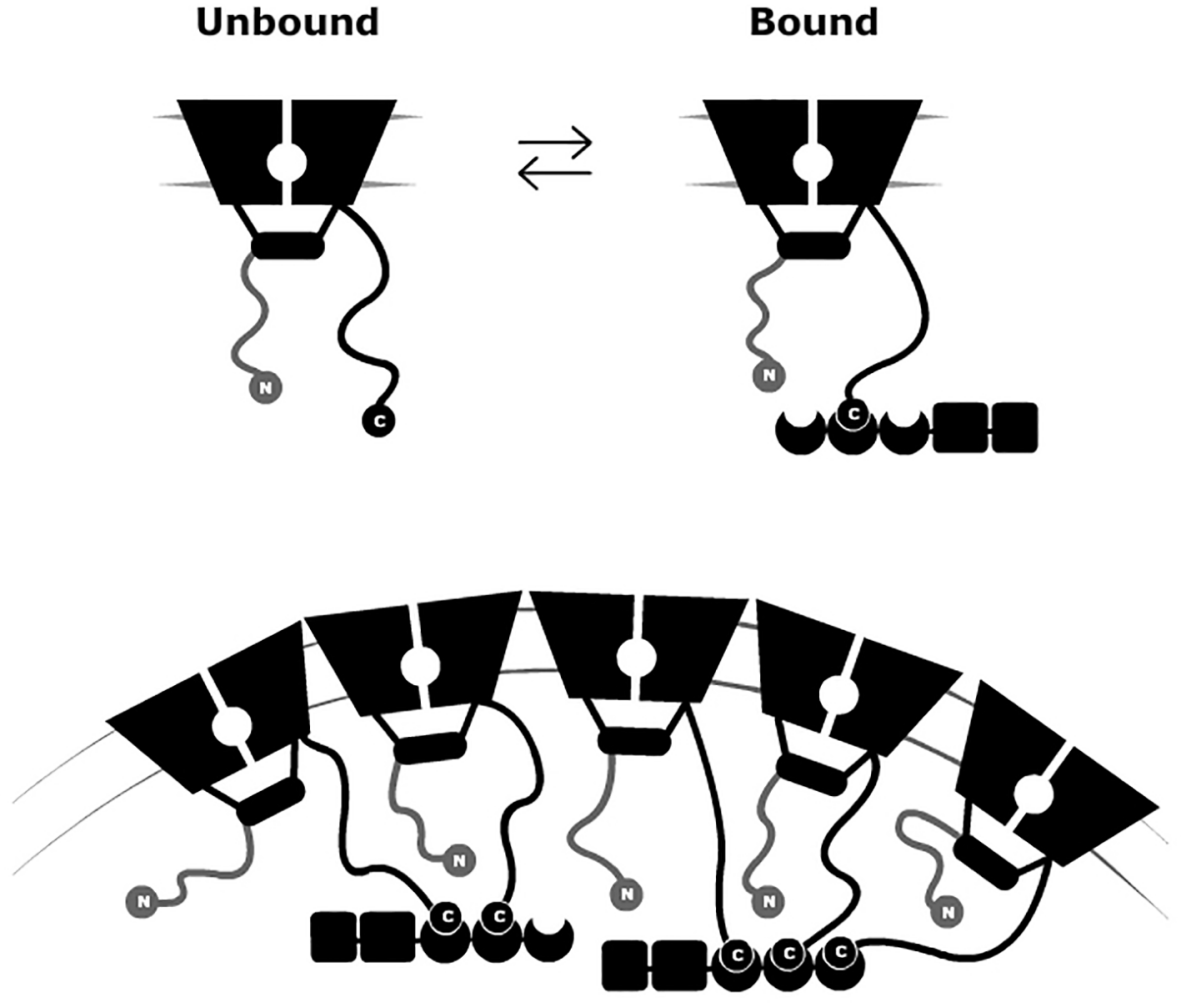
A “ball and chain” mechanism for Kv channel clustering. Schematic representation of the “ball and chain” mechanism for channel binding to PSD-95 (upper panel). In the inter-molecular ‘ball and chain’ binding mechanism, the interaction of the Kv channel with the membrane-associated PSD-95 scaffold protein is precisely timed, as determined by C-terminal chain length, upon binding of the “chain”-tethered peptide “ball” to the PSD-95 PDZ domain(s). Given the stoichiometry of the interaction and the ability of PSD-95 to aggregate, channel clustering results (lower panel). The membrane-embedded portion corresponds to the channel voltage-sensor and pore domains, while the rectangular shape corresponds to the T1 assembly domain. The crescent, box, and rectangular shapes represent the PDZ, SH3, and guanylate kinase-like domains of the PSD-95 protein, respectively. PSD, post-synaptic density.

Members of the *Shaker* Kv channel family were previously discovered to cluster upon channel interaction with the PSD-95 scaffold protein (Kim et al., 1995; Tejedor et al., 1997). This interaction is mediated by PDZ-binding motifs located at the extended C-terminal region of the channel that binds to the PSD-95 PDZ domains. Mutations in the PDZ-binding motif of the prototypical *Drosophila melanogaster Shaker* Kv channel were found to impair both PSD-95 association and clustering. Eliminating this motif resulted in a diffusive channel expression pattern in the membrane of heterologous cells transfected to express the mutant channel and PSD-95 (Kim et al., 1995; Tejedor et al., 1997; Tiffany et al., 2000). In recent years, our lab has revealed the molecular mechanism underlying the *Shaker* Kv channel-PSD-95 interaction (Magidovich et al., 2007; Zandany et al., 2015a). This mechanism is referred to as a ‘ball and chain’ binding mechanism and is depicted in **Figure 2** (upper panel). According to this mechanism, and in analogy to the fast inactivation gating of NaV and Kv channels (Armstrong and Bezanilla, 1977; Armstrong, 1981; Hoshi et al., 1990), the channel’s cytoplasmic C-terminal tail contains an extended intrinsically disordered amino acid “chain” (Magidovich et al., 2007), bearing a conserved PDZ-binding motif (the “ball”) at its tip (Magidovich et al., 2007; Zandany et al., 2015a). The random walk motion of the “chain” serves to search and recruit the PSD-95 scaffold protein partner (**Figure 2** upper panel), in a manner analogous to the role of the extended N-terminal Kv channel tail in regulating fast channel inactivation (Hoshi et al., 1990). In this inactivation process, the stretch comprising the terminal 20 amino acids of the channel, corresponding to the inactivation “ball”, is guided by a random walk search of the attached “chain” for its receptor site in the inner cavity of the open ion conduction pore domain, thus blocking the flow of K^+^ current through the channel. In both “ball and chain” mechanisms, either the N- or C-terminal “chains” belong to the class of entropic chains of intrinsically-disordered proteins (Dunker et al., 2001; Dunker et al., 2002; Uversky and Dunker, 2010) and function as entropic clocks (Dunker et al., 2001) to time channel entry into inactivation or PSD-95 binding, respectively (Hoshi et al., 1990; Zandany et al., 2015a; Zandany et al., 2015b).

Two major criteria support the proposed “ball and chain” mechanism for channel binding to PSD-95 (Zandany et al., 2015a; Zandany et al., 2015b). First, thermodynamic binding analyses showed that the association of the Kv channel C-terminal “chain” peptide with PSD-95 is entropy -controlled in a manner dictated by “chain” length. Second, the association rate constant of the two proteins depends on “chain” length according to a power-law relation, as predicted by polymer chain theory (Zandany et al., 2015a). Direct support for the analogy between the fast inactivation and channel binding to PSD-95 “ball and chain mechanisms” was recently obtained using a “chain”-level chimeric channel approach, where it was shown that different alternatively spliced and intrinsically-disordered N- and C-terminal “chain” variants were able to replace one another in the corresponding fast inactivation or PSD-95 binding processes (Lewin et al., 2019). Furthermore, the swapped “chains” affected the relevant fast inactivation or PSD-95 binding processes in a length-dependent manner, as predicted by the random flight “chain” theory underlying the “ball and chain” mechanism (Lewin et al., 2019).

**Figure 3:**
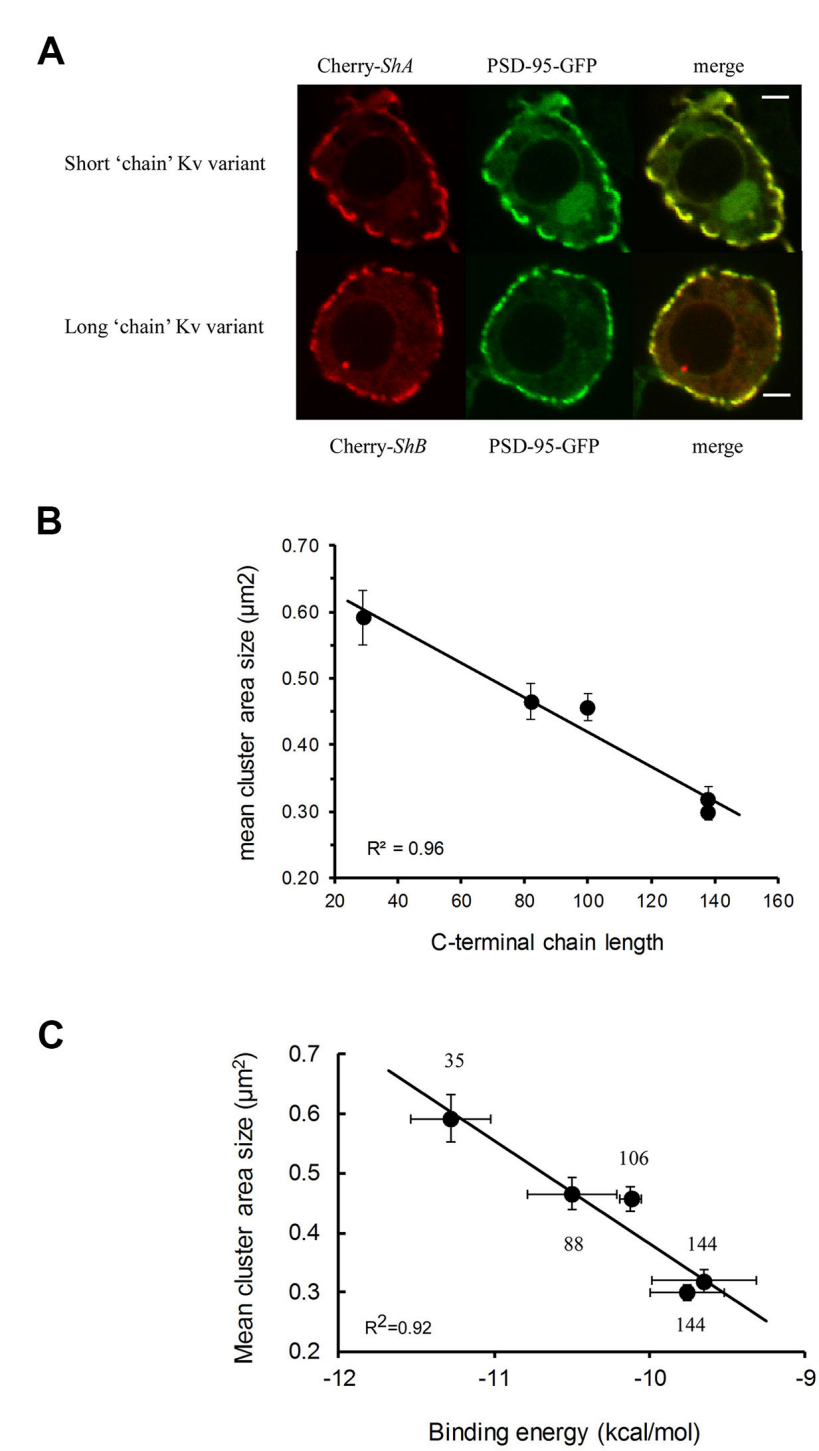
Molecular and cellular correlates in PSD-95–mediated Kv channel clustering. **(A)** Typical confocal microscopy analysis of *Drosophila* Schneider S2 cells co-expressing PSD-95–GFP and either the short or long native (alternatively-spliced) Kv channel “chain” length variants. For each cell, three images are shown, with the red channel-associated and green PSD-95-associated fluorescence signals presented in the left and middle columns, respectively. The merged image is shown in the right column. Scale bars correspond to 2 μ;m. Numbers next to each channel notation indicate C-terminal amino acid “chain” length (adopted with permission from Zandany et al., 2015a). **(B)** Dependence of the mean mega-cluster area size of different Kv channel “chain” length variants on the C-terminal “chain” length. The solid curve corresponds to linear regression with an *R^2^* value of 0.96. **(C)** Correlation plot relating the mean cluster area size of the different Kv channel “chain” length variants, as supported by PSD-95, and the binding affinity of the different variants to PSD-95. The solid curve represents linear regression between the compared quantities (*R*^2^^=^ 0.92). It is possible that such a linear correlation breaks down for too short “chains” due to steric considerations stemming from the inability of multiple Kv channel molecules to bind PSD-95 when the “chain” is too short. Numbers next to each channel notation indicate C-terminal “chain” length in terms of amino acid numbers. PSD, post-synaptic density.

What is the relevance of the molecular “ball and chain” mechanism to cellular *Shaker* channel clustering (**Figure 2**, lower panel)? Clearly, one important aspect is the stoichiometry of the interaction involving the three PDZ domains of PSD-95 and the four “chains” of the tetrameric Kv channel (Gomperts, 1996). Furthermore, the ability of PSD-95 protein to self-aggregate may also help rationalize how multiple ion channels and/or PSD-95 molecules realize close proximity (Hsueh et al., 1997; Ghosh et al., 2018). Given the “chain”-length dependence of the Kv channel-PSD-95 interaction, combined with stoichiometry considerations, one can ask whether Kv channel “chain” length affects attributes of channel clustering, such as cell surface expression, cluster area size or density. These questions are particularly interesting given that alternative splicing of the Kv channel gene produces Kv channel variants exhibiting distinct “chain” lengths yet which present identical terminal “ball” motifs (Kamb et al., 1988; Schwarz et al., 1988). It has been shown that the level of PSD-95-mediated channel membrane surface expression is dependent on “chain” length, with the shorter “chain” length native *Shaker* channel splice variant exhibiting higher affinity to PSD-95 and higher levels of expression, as compared to the longer, lower affinity native channel variant (Zandany et al., 2015a). The same variants further exhibited differences in cluster area size, with the short “chain” variant presenting clusters covering larger areas (**Figure 3A**) (Zandany et al., 2015a). Moreover, confocal imaging analyses of embryonic *Drosophila* Schneider cells transfected to express PSD-95, together with a series of Kv channel native and artificial “chain”-length variants, revealed “chain length”-dependence of Kv channel cluster area size (Lewin et al., 2019). Specifically, the shorter the Kv channel C-terminal “chain”, the larger was the cluster area size observed (**Figure 3B**) (Lewin et al., 2019). Furthermore, the cluster area size of any Kv channel-PSD-95 protein pair was found to linearly correlate with the observed affinity between the two proteins (**Figure 3C**). These results carry important consequences for understanding the regulation of Kv channel clustering. Fine-tuning of channel clustering may not only be achieved by differences in spatial-temporal expression of the PSD-95-related affinity variants (Mottes and Iverson, 1995; Ingleby et al., 2009) but also can result from heterologous subunit assembly (Isacoff et al., 1990; McCormack et al., 1990). Different combinations of long and short C-terminal “chain” variants may thus lead to the appearance of channels with a repertoire of affinities towards PSD-95 and hence, possibly to distinct channel cluster area sizes and densities. Such regulation is expected to have a profound implication on electrical signaling. Taken together, the results summarized in **Figure 3** reveal the cellular correlates of the molecular “ball and chain” mechanism concerning channel clustering. Kv channel “chain” length not only affects affinity to the PSD-95 scaffold protein but further determines Kv channel surface expression and cluster area size. Given the extended intrinsically-disordered nature of this random “chain”, this emphasizes the entropy-based regulation mode of Kv channel clustering, which mirrors the thermodynamic entropy signature of the preceding Kv channel-PSD-95 molecular binding step (Zandany et al., 2015b).

Nevertheless, several questions related to the regulation of *Shaker* Kv channel clustering await answers. What regulation strategies are employed at the “chain” and “ball” levels to control channel binding to PSD-95, and as such, Kv channel clustering? Does Kv channel “chain” length affect ion channel density? Does the C-terminal “chain” play an active or a passive role in channel clustering? Does crosstalk exist between the inactivation and clustering “ball and chain” mechanisms? Is “chain” length a primary factor that determines the binding specificity of PSD-95 towards its multiple membrane protein partners, or post-translational modifications are also involved? While these questions remain to be answered, the “ball and chain” description of Kv channel binding to scaffold proteins provides a simple mechanistic framework for studying Kv channel clustering. It offers a reference framework to analyze, compare, and interpret data and allows for rationalizing different regulation strategies for channel clustering.

## Future Challenges and Directions

While a known mechanism for the ion channel-scaffold protein interaction is definitely valuable for answering the questions posed above, it is not enough. We still need a reliable quantitative approach to ion channel clustering that can presently only be achieved *via* high-resolution imaging of channel clustering. In the past, ion channel clustering was usually studied using conventional confocal imaging light microscopy that allows for resolutions up to 250 nm in the lateral direction. At such low resolution, quantitative assessment of channel clustering yields only limited basic information on surface expression and mega-cluster area size (Zandany et al, 2015a; Lewin et al., 2019). Recent advances in imaging techniques, in particular, in super-resolution imaging methodologies, offer improved spatial resolution reaching 30 nm, a value that may enable the further studying of clustering at the single-molecule level (Sieben et al., 2018; Schermelleh et al., 2019). Such methodologies, particularly single-molecule localization methods (SML), will further enable evaluating important quantitative attributes of channel clustering, such as ion channel densities within cluster sites, number of clusters per membrane area and average number of molecules in a cluster, to mention only a few. It may further enable understanding the various regulation strategies that may affect ion channel density. The premise of such an approach was elegantly demonstrated in a recent paper by Shapiro and colleagues studying ion channel clustering using stochastic optical reconstruction SML microscopy (STORM) (Zhang et al., 2016). In this study, the authors demonstrated scaffold protein (the AKAP protein)-mediated cluster formation involving direct association of different ion channel types. The analysis reported the distribution of clusters according to area size and the relative proximity of the clusters. Their results provided unexpected and novel insight into the role of coupling among different channel types for electrical signaling. As in all reported cases of ion channel coupling due to channel clustering, the channel determinants responsible for such potentially inter-molecular allosteric coupling remain to be identified (Zhang et al., 2016).

Additional important insight into ion channel clustering can be obtained by employing crystallographic and X-ray scattering analyses. Relying on these methods, combined with thermodynamic analyses, Rodzli and colleagues recently showed that the isolated C-terminal peptide of the Kir 2.1 channel, when fused to the first two PDZ domains of PSD-95, forms a cubic-packed, highly organized oligomeric scaffold (Rodzli et al., 2019). These data suggest that ‘chain’ binding to PSD-95 provides a switch with which to initiate channel clustering, probably *via* self-assembly of repeating scaffold units. It would be interesting to examine how changes in length and composition of the peptide “chain” would impact the properties of this compound scaffold.

## Concluding Remarks

In the current review, using the concrete example of PSD-95-mediated *Shaker* Kv channel clustering, we emphasized that insight into cellular ion channel clustering can be obtained when the molecular mechanism controlling ion channel-scaffold protein interaction is known. Identifying the interacting scaffold protein partners responsible for active ion channel clustering is only the first step towards understanding the clustering process. Knowing the molecular mechanism underlying this interaction, combined with super-resolution confocal microscopy, in particular, single-molecule localization microscopy that allows direct observation of channel clustering at the molecular level, may serve to bridge the molecular and cellular gap in understanding channel clustering. This strategy may further provide a framework to analyze pressing questions regarding the specific clustering of the ion channel under study, in particular, those related to the manner(s) by which the ion channel clustering process is regulated to affect ion channel density. Given the seminal importance of ion channel density in affecting action potential conduction properties, brought about by changes in ionic current densities, ion channel gating properties and/or membrane capacitance, efforts to reveal such mechanisms are paramount and are expected to shed more light on the hidden dimension of ion channel density regulation.

## Author Contributions

VN and OY wrote the manuscript.

## Conflict of Interest

The authors declare that the research was conducted in the absence of any commercial or financial relationships that could be construed as a potential conflict of interest.
