# Supplementary material for: Bridging the Molecular-Cellular Gap in Understanding Ion Channel Clustering
Source: Front Pharmacol. 2020 Jan 29;10:1644. doi: 10.3389/fphar.2019.01644 (PMC7000920; doi:10.3389/fphar.2019.01644)
Supplement: Supplementary file 1 [file DataSheet_1.docx]

1. ***PSD:***

**Kir2 channels:**

- Leonoudakis, D., Mailliard, W., Wingerd, K., Clegg, D., and Vandenberg, C. (2001). Inward rectifier potassium channel Kir2.2 is associated with synapse-associated protein SAP97. *J.Cell Sci.* 114, 987–998.
- Hoffmann, B., Klöcker, N., Benndorf, K., and Biskup, C. (2015). Visualization of the dynamics of PSD-95 and Kir2.1 interaction by fluorescence lifetime-based resonance energy transfer imaging. Medical Photonics 27, 70–82. doi:[10.1016/j.medpho.2014.11.001](https://doi.org/10.1016/j.medpho.2014.11.001).

**NMDA and AMPA receptors:**

- Chen, X., Levy, J. M., Hou, A., Winters, C., Azzam, R., Sousa, A. A., et al. (2015). PSD-95 family MAGUKs are essential for anchoring AMPA and NMDA receptor complexes at the postsynaptic density. Proc. Natl. Acad. Sci. U.S.A. 112, E6983-6992. doi:[10.1073/pnas.1517045112](https://doi.org/10.1073/pnas.1517045112).
- Scheefhals, N., and MacGillavry, H. D. (2018). Functional organization of postsynaptic glutamate receptors. Mol. Cell. Neurosci. 91, 82–94. doi:[10.1016/j.mcn.2018.05.002](https://doi.org/10.1016/j.mcn.2018.05.002).

**Glycin and GABA(A) receptors:**

- Patrizio, A., Renner, M., Pizzarelli, R., Triller, A., and Specht, C. G. (2017). Alpha subunit-dependent glycine receptor clustering and regulation of synaptic receptor numbers. Sci. Rep. 7, 10899. doi:[10.1038/s41598-017-11264-3](https://doi.org/10.1038/s41598-017-11264-3).
- Kasaragod, V. B., and Schindelin, H. (2018). Structure–Function Relationships of Glycine and GABAA Receptors and Their Interplay With the Scaffolding Protein Gephyrin. Front. Mol. Neurosci. 11. doi:[10.3389/fnmol.2018.00317](https://doi.org/10.3389/fnmol.2018.00317).
- Tretter, V., Jacob, T. C., Mukherjee, J., Fritschy, J.-M., Pangalos, M. N., and Moss, S. J. (2008). The Clustering of GABAA Receptor Subtypes at Inhibitory Synapses is Facilitated via the Direct Binding of Receptor α2 Subunits to Gephyrin. J. Neurosci. 28, 1356–1365. doi:[10.1523/JNEUROSCI.5050-07.2008](https://doi.org/10.1523/JNEUROSCI.5050-07.2008).

1. ***AIS:***

**Kv1 channels:**

- Leterrier, C. (2018). The Axon Initial Segment: An Updated Viewpoint. J. Neurosci. 38, 2135–2145. doi:[10.1523/JNEUROSCI.1922-17.2018](https://doi.org/10.1523/JNEUROSCI.1922-17.2018).
- Kole, M. H. P., Letzkus, J. J., and Stuart, G. J. (2007). Axon initial segment Kv1 channels control axonal action potential waveform and synaptic efficacy. Neuron 55, 633–647. doi:10.1016/j.neuron.2007.07.031.
- Ogawa, Y., Horresh, I., Trimmer, J. S., Bredt, D. S., Peles, E., and Rasband, M. N. (2008). Postsynaptic density-93 clusters Kv1 channels at axon initial segments independently of Caspr2. J. Neurosci. 28, 5731–5739. doi:[10.1523/JNEUROSCI.4431-07.2008](https://doi.org/10.1523/JNEUROSCI.4431-07.2008).
- Traka, M., Goutebroze, L., Denisenko, N., Bessa, M., Nifli, A., Havaki, S., et al. (2003). Association of TAG-1 with Caspr2 is essential for the molecular organization of juxtaparanodal regions of myelinated fibers. J. Cell Biol. 162, 1161–1172. doi:[10.1083/jcb.200305078](https://doi.org/10.1083/jcb.200305078).

**Nav channels:**

- Freeman, S. A., Desmazières, A., Fricker, D., Lubetzki, C., and Sol-Foulon, N. (2016). Mechanisms of sodium channel clustering and its influence on axonal impulse conduction. Cell. Mol. Life Sci. 73, 723–735. doi:[10.1007/s00018-015-2081-1](https://doi.org/10.1007/s00018-015-2081-1).
- Garrido, J. J., Giraud, P., Carlier, E., Fernandes, F., Moussif, A., Fache, M.-P., et al. (2003). A Targeting Motif Involved in Sodium Channel Clustering at the Axonal Initial Segment. Science 300, 2091–2094. doi:[10.1126/science.1085167](https://doi.org/10.1126/science.1085167).
- Lemaillet, G., Walker, B., and Lambert, S. (2003). Identification of a Conserved Ankyrin-binding Motif in the Family of Sodium Channel α Subunits. J. Biol. Chem. 278, 27333–27339. doi:[10.1074/jbc.M303327200](https://doi.org/10.1074/jbc.M303327200).
- Misonou, H. (2018). Precise localizations of voltage-gated sodium and potassium channels in neurons. Dev. Neurobiol. 78, 271–282. doi:[10.1002/dneu.22565](https://doi.org/10.1002/dneu.22565).
- Zhou, D., Lambert, S., Malen, P. L., Carpenter, S., Boland, L. M., and Bennett, V. (1998). AnkyrinG is required for clustering of voltage-gated Na channels at axon initial segments and for normal action potential firing. J. Cell Biol. 143, 1295–1304. doi:[10.1083/jcb.143.5.1295](https://doi.org/10.1083/jcb.143.5.1295).

**Kv2 channels:**

- Jensen, C. S., Watanabe, S., Stas, J. I., Klaphaak, J., Yamane, A., Schmitt, N., et al. (2017). Trafficking of Kv2.1 Channels to the Axon Initial Segment by a Novel Nonconventional Secretory Pathway. J Neurosci 37, 11523–11536. doi:[10.1523/JNEUROSCI.3510-16.2017](https://doi.org/10.1523/JNEUROSCI.3510-16.2017).
- King, A. N., Manning, C. F., and Trimmer, J. S. (2014). A unique ion channel clustering domain on the axon initial segment of mammalian neurons. J. Comp. Neurol. 522, 2594–2608. doi:[10.1002/cne.23551](https://doi.org/10.1002/cne.23551).

**KCNQ channels:**

- Klinger, F., Gould, G., Boehm, S., and Shapiro, M. S. (2011). Distribution of M-channel subunits KCNQ2 and KCNQ3 in rat hippocampus. Neuroimage. 58, 761–769. doi:[10.1016/j.neuroimage.2011.07.003](https://doi.org/10.1016/j.neuroimage.2011.07.003).
- Pan, Z. (2006). A Common Ankyrin-G-Based Mechanism Retains KCNQ and NaV Channels at Electrically Active Domains of the Axon. J. Neurosci. 26, 2599–2613. doi:[10.1523/JNEUROSCI.4314-05.2006](https://doi.org/10.1523/JNEUROSCI.4314-05.2006).

1. **NODES OF THE RANVIER:**

**KCNQ channels:**

- Devaux, J. J., Kleopa, K. A., Cooper, E. C., and Scherer, S. S. (2004). KCNQ2 is a nodal K+ channel. J. Neurosci. 24, 1236–1244. doi:[10.1523/JNEUROSCI.4512-03.2004](https://doi.org/10.1523/JNEUROSCI.4512-03.2004).
- Klinger, F., Gould, G., Boehm, S., and Shapiro, M. S. (2011). Distribution of M-channel subunits KCNQ2 and KCNQ3 in rat hippocampus. Neuroimage. 58, 761–769. doi:[10.1016/j.neuroimage.2011.07.003](https://doi.org/10.1016/j.neuroimage.2011.07.003).s

**NaV channels:**

- Freeman, S. A., Desmazières, A., Fricker, D., Lubetzki, C., and Sol-Foulon, N. (2016). Mechanisms of sodium channel clustering and its influence on axonal impulse conduction. Cell. Mol. Life Sci. 73, 723–735. doi:[10.1007/s00018-015-2081-1](https://doi.org/10.1007/s00018-015-2081-1).
- Lemaillet, G., Walker, B., and Lambert, S. (2003). Identification of a Conserved Ankyrin-binding Motif in the Family of Sodium Channel α Subunits. J. Biol. Chem. 278, 27333–27339. doi:[10.1074/jbc.M303327200](https://doi.org/10.1074/jbc.M303327200).
- Misonou, H. (2018). Precise localizations of voltage-gated sodium and potassium channels in neurons. Dev. Neurobiol. 78, 271–282. doi:[10.1002/dneu.22565](https://doi.org/10.1002/dneu.22565).
- Salzer, J. L. (1997). Clustering sodium channels at the node of Ranvier: close encounters of the axon-glia kind. *Neuron* 18, 843–846. doi:[10.1016/s0896-6273(00)80323-2](https://doi.org/10.1016/s0896-6273(00)80323-2).

**Kv3.1 channels:**

- Xu, M., Cao, R., Xiao, R., Zhu, M. X., and Gu, C. (2007). The axon-dendrite targeting of Kv3 (Shaw) channels is determined by a targeting motif that associates with the T1 domain and ankyrin G. J. Neurosci. 27, 14158–14170. doi:[10.1523/JNEUROSCI.3675-07.2007](https://doi.org/10.1523/JNEUROSCI.3675-07.2007).
- Devaux, J., Alcaraz, G., Grinspan, J., Bennett, V., Joho, R., Crest, M., et al. (2003). Kv3.1b is a novel component of CNS nodes. J. Neurosci. 23, 4509–4518.
- Rasband, M. N. (2010). Clustered K+ channel complexes in axons. Neuroscience Letters 486, 101–106. doi:[10.1016/j.neulet.2010.08.081](https://doi.org/10.1016/j.neulet.2010.08.081).

1. **JUXTAPARANODES:**

**Kv1 channels:**

- Rasband, M. N. (2004). It’s “juxta” potassium channel! J. Neurosci. Res. 76, 749–757. doi:[10.1002/jnr.20073](https://doi.org/10.1002/jnr.20073).
- Rasband, M. N. (2010). Clustered K+ channel complexes in axons. Neurosci. Lett. 486, 101–106. doi:[10.1016/j.neulet.2010.08.081](https://doi.org/10.1016/j.neulet.2010.08.081).
- Ogawa, Y., Oses-Prieto, J., Kim, M. Y., Horresh, I., Peles, E., Burlingame, A. L., et al. (2010). ADAM22, a Kv1 channel-interacting protein, recruits membrane-associated guanylate kinases to juxtaparanodes of myelinated axons. J. Neurosci. 30, 1038–1048. doi:[10.1523/JNEUROSCI.4661-09.2010](https://doi.org/10.1523/JNEUROSCI.4661-09.2010).
- Wang, H., Kunkel, D. D., Martin, T. M., Schwartzkroin, P. A., and Tempel, B. L. (1993). Heteromultimeric K+ channels in terminal and juxtaparanodal regions of neurons. Nature 365, 75–79. doi:[10.1038/365075a0](https://doi.org/10.1038/365075a0).
- Poliak, S., Salomon, D., Elhanany, H., Sabanay, H., Kiernan, B., Pevny, L., et al. (2003). Juxtaparanodal clustering of Shaker-like K+ channels in myelinated axons depends on Caspr2 and TAG-1. J. Cell Biol. 162, 1149–1160. doi:[10.1083/jcb.200305018](https://doi.org/10.1083/jcb.200305018).
- Arancibia-Carcamo, I. L., and Attwell, D. (2014). The node of Ranvier in CNS pathology. Acta Neuropathol. 128, 161–175. doi:[10.1007/s00401-014-1305-z](https://doi.org/10.1007/s00401-014-1305-z).
- Rasband, M. N., Park, E. W., Zhen, D., Arbuckle, M. I., Poliak, S., Peles, E., et al. (2002). Clustering of neuronal potassium channels is independent of their interaction with PSD-95. J. Cell Biol. 159, 663–672. doi:[10.1083/jcb.200206024](https://doi.org/10.1083/jcb.200206024).

1. **ACTIVE ZONE:**

**CaV channels:**

- Gundelfinger, E. D., and Fejtova, A. (2012). Molecular organization and plasticity of the cytomatrix at the active zone. Curr. Opin. Neurobiol. 22, 423–430. doi:[10.1016/j.conb.2011.10.005](https://doi.org/10.1016/j.conb.2011.10.005).
- Kaeser, P. S., Deng, L., Wang, Y., Dulubova, I., Liu, X., Rizo, J., et al. (2011). RIM proteins tether Ca2+-channels to presynaptic active zones via a direct PDZ-domain interaction. Cell 144, 282–295. doi:[10.1016/j.cell.2010.12.029](https://doi.org/10.1016/j.cell.2010.12.029).
- Krinner, S., Butola, T., Jung, S., Wichmann, C., and Moser, T. (2017). RIM-Binding Protein 2 Promotes a Large Number of CaV1.3 Ca2+-Channels and Contributes to Fast Synaptic Vesicle Replenishment at Hair Cell Active Zones. Front. Cell Neurosci. 11. doi:[10.3389/fncel.2017.00334](https://doi.org/10.3389/fncel.2017.00334).
- Miki, T., Kaufmann, W. A., Malagon, G., Gomez, L., Tabuchi, K., Watanabe, M., et al. (2017). Numbers of presynaptic Ca2+ channel clusters match those of functionally defined vesicular docking sites in single central synapses. Proc. Natl. Acad. Sci. U.S.A. 114, E5246–E5255. doi:[10.1073/pnas.1704470114](https://doi.org/10.1073/pnas.1704470114).
- Südhof, T. C. (2012). The presynaptic active zone. Neuron 75, 11–25. doi:[10.1016/j.neuron.2012.06.012](https://doi.org/10.1016/j.neuron.2012.06.012).

**Basket cell terminals:**

- Kole, M. J., Qian, J., Waase, M. P., Klassen, T. L., Chen, T. T., Augustine, G. J., et al. (2015). Selective Loss of Presynaptic Potassium Channel Clusters at the Cerebellar Basket Cell Terminal Pinceau in Adam11 Mutants Reveals Their Role in Ephaptic Control of Purkinje Cell Firing. J.Neurosci. 35, 11433–11444. doi:[10.1523/JNEUROSCI.1346-15.2015](https://doi.org/10.1523/JNEUROSCI.1346-15.2015).
- Rasband, M. N. (2010). Clustered K+ channel complexes in axons. Neurosci. Lett. 486, 101–106. doi:[10.1016/j.neulet.2010.08.081](https://doi.org/10.1016/j.neulet.2010.08.081).
